# The Effects of Cadmium Toxicity

**DOI:** 10.3390/ijerph17113782

**Published:** 2020-05-26

**Authors:** Giuseppe Genchi, Maria Stefania Sinicropi, Graziantonio Lauria, Alessia Carocci, Alessia Catalano

**Affiliations:** 1 Dipartimento di Farmacia e Scienze della Salute e della Nutrizione, Università della Calabria, 87036 Arcavacata di Rende (Cosenza), Italy; giuseppe.genchi@unical.it (G.G.); glauria@unical.it (G.L.); 2 Dipartimento di Farmacia-Scienze del Farmaco, Università degli Studi di Bari “A. Moro”, 70125 Bari, Italy; alessia.catalano@uniba.it

**Keywords:** antioxidant, apoptosis, cadmium, cancer, chelators, metallothioneins, phytoremediation, damage of mitochondria

## Abstract

Cadmium (Cd) is a toxic non-essential transition metal that poses a health risk for both humans and animals. It is naturally occurring in the environment as a pollutant that is derived from agricultural and industrial sources. Exposure to cadmium primarily occurs through the ingestion of contaminated food and water and, to a significant extent, through inhalation and cigarette smoking. Cadmium accumulates in plants and animals with a long half-life of about 25–30 years. Epidemiological data suggest that occupational and environmental cadmium exposure may be related to various types of cancer, including breast, lung, prostate, nasopharynx, pancreas, and kidney cancers. It has been also demonstrated that environmental cadmium may be a risk factor for osteoporosis. The liver and kidneys are extremely sensitive to cadmium’s toxic effects. This may be due to the ability of these tissues to synthesize metallothioneins (MT), which are Cd-inducible proteins that protect the cell by tightly binding the toxic cadmium ions. The oxidative stress induced by this xenobiotic may be one of the mechanisms responsible for several liver and kidney diseases. Mitochondria damage is highly plausible given that these organelles play a crucial role in the formation of ROS (reactive oxygen species) and are known to be among the key intracellular targets for cadmium. When mitochondria become dysfunctional after exposure to Cd, they produce less energy (ATP) and more ROS. Recent studies show that cadmium induces various epigenetic changes in mammalian cells, both in vivo and in vitro, causing pathogenic risks and the development of various types of cancers. The epigenetics present themselves as chemical modifications of DNA and histones that alter the chromatin without changing the sequence of the DNA nucleotide. DNA methyltransferase, histone acetyltransferase, histone deacetylase and histone methyltransferase, and micro RNA are involved in the epigenetic changes. Recently, investigations of the capability of sunflower (*Helianthus annuus* L.), Indian mustard (*Brassica juncea*), and river red gum (*Eucalyptus camaldulensis*) to remove cadmium from polluted soil and water have been carried out. Moreover, nanoparticles of TiO_2_ and Al_2_O_3_ have been used to efficiently remove cadmium from wastewater and soil. Finally, microbial fermentation has been studied as a promising method for removing cadmium from food. This review provides an update on the effects of Cd exposure on human health, focusing on the cellular and molecular alterations involved.

## 1. Introduction

Cadmium (Cd), alongside arsenic, lead, mercury, and chromium, is a heavy metal that does not have a physiological function and is often considered a toxicant [1,2,3,4,5,6,7]. Many different forms of exposure to cadmium have been shown over the past century, with cadmium being present in the environment as a result of many human activities [8]. The constant sources of cadmium contamination are related to its application in industry as a corrosive reagent, as well as its use as a stabilizer in PVC products, color pigments, and Ni-Cd batteries [9]. In areas with contaminated soils, house dust is a potential route for cadmium exposure [10]. Anthropogenic sources of Cd in the environment derive from copper and nickel smelting and refining, fossil fuel combustion, and the use of phosphate fertilizers. Cadmium is also present as a pollutant in non-ferrous metal smelters and the recycling of electronic waste. Volcanic activity, the gradual process of erosion and abrasion of rocks and soil, and forest fires are among the reasons for the increase in Cd concentrations in the living environment (atmosphere, soil, and water); even zinc, lead, and copper mines contribute to the release of this metal into the atmosphere, resulting in the contamination of soil [11]. The absorption of Cd takes place mainly through the respiratory tract and to a smaller extent via the gastro-intestinal tract, while skin absorption is relatively rare. When cadmium enters the body, it is transported into the bloodstream via erythrocytes and albumin and is then accumulated in the kidneys [12], liver, and gut [13]. Cadmium excretion from the body is slow and occurs via the kidneys, urine, saliva, and milk during lactation. In humans, Cd exposure can result in a variety of adverse effects, such as renal and hepatic dysfunction, pulmonary edema, testicular damage, osteomalacia, and damage to the adrenals and hemopoietic system [14]. An association between Cd exposure markers (blood and urine) and coronary heart disease, stroke, peripheral artery disease, and atherogenic changes in lipid profile was also observed [15]. In addition to its cytotoxic effects that could lead to apoptotic or necrotic events, cadmium is a proven human carcinogen (group I of International Agency for Research on Cancer classification) [14,16]. Occupational or environmental cadmium exposure has been related to lung, breast, prostate, pancreas, urinary bladder, and nasopharynx cancers [17]. The generation of reactive oxygen species, accumulation of Ca^2+^, upregulation of caspase-3, downregulation of bcl-2, and deficiency of p-53 lead to Cd-induced apoptosis. Recently, it has also been demonstrated that cadmium arsenite in yeast cells may interfere with the folding of nascent proteins, which reduces cellular viability and is probably responsible for various pathological conditions, such as neurodegenerative diseases and age-related disorders, and Alzheimer’s and Parkinson’s diseases [18,19]. Additionally, exposure to cadmium is recognized as one of the risk factors for osteoporosis, although critical exposure levels and the exact mechanisms are still unknown [20], and a relationship between prenatal Cd exposure and cognitive and kidneys development in fetuses has been demonstrated [21,22]. Cadmium may interfere with the activity of antioxidant enzymes, such as catalase, manganese superoxide dismutase, and copper-zinc superoxide dismutase. Metallothionein is a zinc-concentrating protein that can act as a free-radical scavenger. Cells containing metallothioneins are resistant to cadmium toxicity, while cells that cannot synthesize metallothioneins are sensitive to its intoxication [23]. Metallothionein expression determines the choice between apoptosis and necrosis in Cd-induced toxicity [24]. In this review, we present recent literature regarding cellular and molecular alterations caused by exposure to Cd, focusing first on its effects on mitochondria. Cadmium acts on mitochondria by inducing oxidative stress and generating ROS, activating apoptosis, mutating mtDNA, altering gene expression, inhibiting respiratory chain complexes, reducing ATP synthesis, and finally altering the inner mitochondrial permeability. This results in the development of numerous human disorders.

## 2. Chemical Form and Properties of Cadmium

Cadmium (Cd; atomic number 48, atomic weight 112.41) belongs to group XII of the periodic table of chemical elements. This soft, silvery-white metal is chemically similar to zinc and mercury in its physical and chemical properties (Table 1). Cadmium’s atomic weight results from a mixture of eight stable isotopes. It is a post-transition metal and possesses two electrons in the s orbital and a complete d orbital. Moreover, as for zinc, the soft, malleable, ductile cadmium prefers the oxidation state of +2 in most of its compounds. Cd is resistant to corrosion and is used as a protective plate; it is water-insoluble and is not flammable. Cd burns in air, forming cadmium oxide. Hydrochloric, sulfuric, and nitric acids dissolve cadmium by forming cadmium chloride, cadmium sulfate, and cadmium nitrate, respectively. Cadmium is also used in control rods for nuclear reactors, acting as a neutron poison to control neutron flux in nuclear fission.

Cadmium is found in nature at low concentrations, mainly with the sulfide ores of zinc, lead, and copper. Cadmium ores are not abundant, but Cd may be found in most zinc ores given its isomorphic substitution to zinc. However, due to its widespread occurrence, cadmium is found in measurable amounts in food, drink, and breath [1,25]. Human activities, such as the combustion of fossil fuels, as well as leachate generated from landfill sites, agricultural land, and mining waste, especially from zinc and lead mines, contribute to cadmium contamination in the environment [26]. Cadmium is used as a pigment, stabilizer of PVC and alloys, and in the electroplating used to protect steel from corrosion [15,25,27]. Cadmium compounds are often used in coloring borosilicate glass and in lampworking. Cadmium is also produced in the manufacturing of Ni-Cd batteries [9]. However, nowadays, the use of cadmium is decreasing because of its toxicity, and Ni-Cd batteries have been replaced with nickel-metal hydride and lithium-ion batteries. One of its new uses is as cadmium telluride semiconducting solar panels and infrared optical windows. Cadmium oxide is an n-type semiconductor [28]. It is used in cadmium plating baths, electrodes for storage batteries, catalysts, and ceramic glazes. The major use for cadmium oxide is as an ingredient for electroplating baths and pigments. Cadmium oxide and cadmium telluride in the form of thin films have been used in applications such as photodiodes, phototransistors, photovoltaic solar cells, transparent electrodes, and anti-reflection coatings [29].

Even if cadmium has no known biological function in higher organisms, a Cd-dependent carbonic anhydrase (CDCA1) has been found in the marine diatom unicellular microalgae *Thalassiosira weissflogii*. These diatoms live in an environment under ambient conditions of zinc limitation, and cadmium replaces zinc in the active site of the enzyme as a catalytic metallic ion [30].

## 3. Exposure to Cadmium and Toxicity

Cadmium is a pollutant introduced in the environment as a result of the rapid development of industries and modern technologies [31]. It is absorbed in high quantities from water, food, and air contaminations. High concentrations of Cd are present in crustaceans, bivalve mollusks, oysters, cephalopods, and crabs; it is also found in offal products, such as liver and kidney, in oil seeds, cocoa beans, and certain wild mushrooms [12]. Foods derived from plants, depending on the level of soil contamination, generally contain higher concentrations of Cd than meat, egg, milk, and dairy products. Among these, rice and wheat, green leafy vegetables, potatoes, carrot, and celery can contain higher concentrations of the metal than other food from plants. Vegetarians and shellfish consumers may be exposed to a higher cadmium intake than omnivores [32]. One of the major routes for cadmium exposure in humans is through rice consumption [33]. For this reason, flooding rice fields at harvest time is recommended as a water management strategy for reducing Cd accumulation in rice, especially in Japan, in areas where there is soil contamination of Cd; this strategy may, however, increase As accumulation in rice [34,35]. Cd presents specific hydrochemical characteristics, causing its potential mobility in groundwater. At near neutral pH (<6.5) it remains in solution, in contrast to the typical fixation of other heavy metals [36]. Thus, heavy metal contamination of both drinking water and rivers with Cd may occur especially in areas near factories or mines [37]. Another environmental source of Cd exposure in the general population is tobacco use [38]. Cadmium exposure from smoking cigarettes may vary depending on the type of cigarettes. A cigarette contains 1–2 μg Cd; a person smoking 20 cigarettes per day will absorb about 1 μg Cd. About 10% of Cd is inhaled with a 40%–50% absorption rate in the lung. Inhaled cadmium is linked to smoking-related respiratory diseases, such as pulmonary disease and lung cancer. Mona et al. found a significant increase of Cd in the serum and urine of smokers compared to non-exposed smokers [39]. They found a significant increase in bone pain percentage in smokers compared to non-exposed smokers (95% vs. 37.7%) and they concluded that long-term exposure to Cd may have an osteotoxic effect, resulting in bone tissue loss.

Cadmium, which used extensively in electroplating, is also found in some industrial paints, and represents a hazard when sprayed. The atmospheric deposition of airborne cadmium, mining, and the use of certain sources of fertilizers containing cadmium phosphate and sewage sludge on farms may lead to soil contamination and increased absorption by crops and vegetables for human consumption.

Environmental exposure to cadmium was a serious problem in Toyama Prefecture (Japan), starting from 1912, where many people consumed rice grown in Cd-contaminated irrigation water. Cadmium was released into Jinzu River basin by a zinc mine, which was sued for the damage [40,41]. It was the outbreak of Itai-Itai disease, which is characterized by severe pains in people with spine and joint injuries. The Itai-Itai patients showed a wide range of symptoms, such as a low grade of bone mineralization, severe skeletal decalcification, a high rate of fractures and distortion of the long bones, osteomalacia, intense bone pain, and osteoporosis, which is characterized by low bone mass and micro-architectural deterioration of bone tissue. Other complications included coughing, anemia, and kidney failure, leading to death. Workers can be exposed to Cd in the air due to smelting and refining metals; people that make Cd products, such as batteries, coating, or plastics can also be exposed to Cd, such as workers involved in soldering or welding metals containing cadmium. Artists who work with cadmium pigments, which are commonly used in yellows, oranges, and reds (cadmium sulfide and cadmium sulfoselenide) can easily accidentally ingest dangerous amounts of these paint pigments. Claude Monet and Vincent Van Gogh first used brilliant cadmium yellow, orange, and red. The greatest use is in the industrial coloring of plastic, which must resist processing or service temperatures up to 3000 °C. The color fastness or permanence of cadmium requires protection from its tendency to form carbonate salts when exposed to air. Nowadays, cadmium dyes have been partially replaced by azo dyes.

Low levels exposure to Cd may lead to damage to the kidneys, liver, skeletal system, and cardiovascular system, as well as to deterioration of sight and hearing. Along with strong teratogenic and mutagenic effects related to cadmium, it also shows adverse effects at low doses on both human male and female reproduction and affects pregnancy or its outcome [42]. This is due to changes in the expression of many genes in the embryo, leading to the aberrant methylation of them within the placenta and the embryo. The epigenetic modification patterns induced by cadmium have been linked to their ability to easily bind to thiols with depletion of the methyl donor S-adenosyl methionine, resulting in methylome alterations, and subsequently, to alteration of DNA methyltransferase activity. This may cause placental and fetal development disorders [43].

Cadmium induces the alteration of steroidogenesis, disorders of the menstrual cycle and reproductive hormones, delay in puberty and menarche, pregnancy loss, premature birth, and reduced birth weight [26]. Cadmium was also negatively related to bone mineral density in postmenopausal women. Women have a higher cadmium body burden than men, reflecting a higher concentration of cadmium in the blood, urine, and kidneys. The intestinal cadmium absorption increases with a depleted body iron store and with overt iron deficiency, conditions that are prevalent in women of fertile age [44]. Recently, the potential involvement of cadmium’s vascular pathology and the risk of a subarachnoid hemorrhage has been studied [45]. Cd may interact with different hormonal signaling pathways and is considered an endocrine disruptor that may bind estrogen receptor alpha and affect signaling transduction along estrogen and MAPK signaling pathways. Low doses of Cd affect the cardiovascular system [46]. Epidemiological studies showed that exposure to Cd may promote the development of musculoskeletal diseases, such as osteoporosis, rheumatoid arthritis (RA), and osteoarthritis (OA) [47].

**Figure 1:**
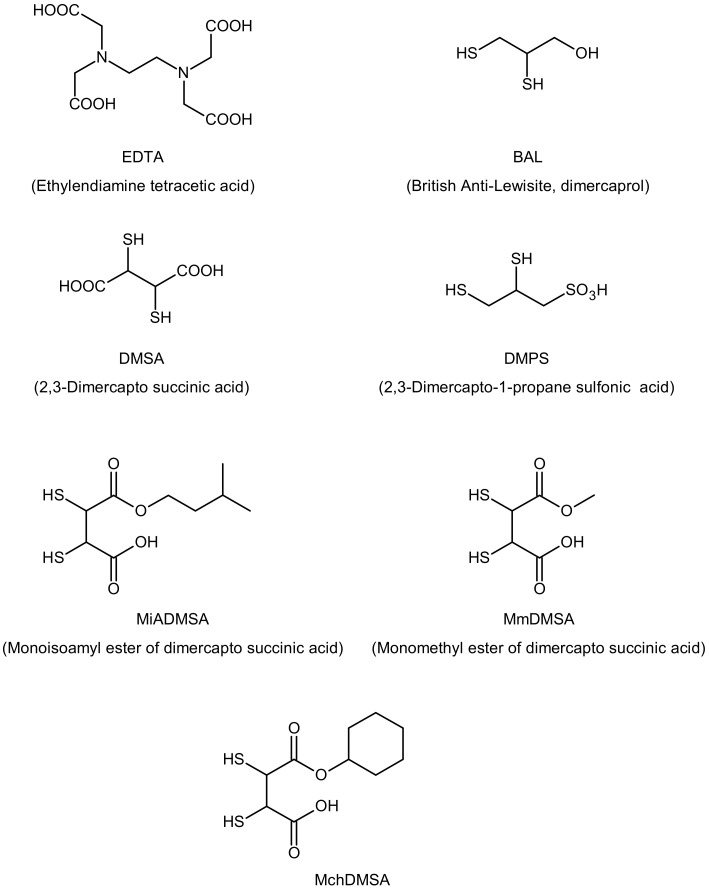
Structures of cadmium chelating agents.

Intoxication by cadmium required immediate medical help. Emesis or a gastric lavage may be beneficial soon after exposure via ingestion. Detoxification of Cd with EDTA (ethylenediamine tetraacetic acid), BAL (British Anti-Lewisite, dimercaprol), DMSA (2,3-dimercapto-succinic acid), and DMPS (2,3-dimercapto-1-propane sulfonic acid) is also possible (Figure 1). BAL and their analogs, DMSA and DMPS, are used as antidotes for heavy metal poisoning [48]. DMPS, unlike DMSA, is transported into the intracellular space, where it works as a chelator. DMSA analogs, such as the esters MmDMSA (monomethyl DMSA), MiADMSA (monoisoamyl DMSA), and MchDMSA (monocyclo hexyl DMSA), are more effective and safe antidotes for heavy metal poisoning compared to DMSA [49]. MiADMSA is a water-soluble, lipophilic chelating agent that can access different endogenous ligands; this drug is preferable to its parent compound [50]. MmDMSA and MchDMSA are lipophilic compounds and can penetrate cells. Combination therapy is an effective route in the management of heavy metal toxicity. Tandon et al. demonstrated that DMPS and NAC (*N*-acetyl cysteine) reduced cadmium-induced hepatic and renal metallothioneins [51]. The optimal effects of chelating agent therapy may be obtained when DMSA and MiADMSA are administered in combination [52].

Moreover, biologically active compounds that occur naturally in plants may be useful for preventing the deleterious health effects resulting from exposure to heavy metals, including cadmium. These exposure compounds include polyphenolic substances, melatonin, carotenoids, quercetine, resveratrol, vitamin E, vitamin C, L-carnitine, and coenzyme Q10 [53,54,55,56]. Among these substances, the polyphenols and antioxidants present in our daily diet in vegetables, fruits, and spices, as well as in drinks like green tea, red wine, and cocoa, are very promising (Table 2) [57,58,59,60,61]. The protective effect of polyphenols derives from their strong antioxidant properties and their ability to chelate cadmium ions. Due to the high affinity of cadmium ions to hydroxyl groups and the ability to increase the expression of intestinal MT, polyphenols decrease the absorption of this xenobiotic metal from the gastrointestinal tract [62]. However, caution must be used with antioxidants in the case of Cd toxicity. Kara et al. demonstrated that both melatonin alone, and melatonin in combination with vitamin E and selenium showed protective effects against cadmium-induced oxidative damage in rat liver [63]. Littlefield and Haas showed that the use of antioxidant vitamin C in the presence of Cd and Ni ions caused the formation of double-stranded DNA breaks and apoptotic cell death, probably due to a Fenton-type reaction in which an antioxidant in the presence of metal ions generates hydroxyl radicals, and consequently, double-stranded DNA breaks [64]. The protective role of β-carotene on cadmium-induced kidney and brain damage has recently been reported [65]. Moreover, royal jelly seems to attenuate Cd-induced nephrotoxicity, probably due to its ability of promoting the nuclear factor erythroid 2–related factor 2 (Nrf2)/antioxidant responsive element (ARE) pathway [66]. Recently, it has been demonstrated that a supplement of glutamate, but not glutamine, significantly alleviated Cd toxicity in hydroponically grown rice plants, probably by suppressing Cd uptake and translocation [67].

Bioelements such as manganese, selenium, zinc, magnesium, calcium, and iron decrease cadmium absorption from the gastrointestinal tract and causes its accumulation in the body. Moreover, some of these elements are present in the active sites of antioxidant enzymes (Cu-Zn-SOD, Cu-Zn superoxide dismutase; Mn-SOD, Mn superoxide dismutase; Fe-CAT, Fe catalase: Se-GPx, Se glutathione peroxidase), protecting from the weakening of antioxidative potential induced by Cd and oxidative stress development [54,55,62,68,69]. Melatonin is a natural antioxidant that may protect biological organisms from oxidative stress. It can prevent metal-mediated damage (iron, copper, cadmium), both in vitro and in metal-loaded animals [70,71].

Recently, in both mammals and plants, transporters originally disclosed for the uptake of essential elements, such as Mn, Zn, and Si, have been used for the uptake of toxic metals, such as Cd and As. A study revealed that OsNramp5, located in the outer membrane of rice roots, is the transporter responsible for the uptake of Mn into rice and shows an affinity for Cd. The knockout of the OsNramp5 gene results in an almost complete loss of Cd absorption into the roots, and consequently, in grains of rice. Mutant rice that was obtained by using ion-beam irradiation has begun to be cultivated in Japan. In this rice, the function of OsNramp5 is lost, and thus Cd is not accumulated [72].

## 4. Cadmium’s Effects on Mitochondria and Cd^2+^-Induced Apoptosis

The mitochondrial respiratory chain plays an essential role in maintaining energy homeostasis through oxidative phosphorylation (OXPHOS), generating energy in the form of adenosine triphosphate (ATP), which the energy necessary for life. Additionally, mitochondria are implicated in the synthesis of amino acids, lipids and phospholipids, ion homeostasis, motility, and apoptosis (programmed death). The number and functions of mitochondria differ in animal tissues and cells relative to energetic needs and in response to physiological or environmental alterations. Malfunctioning mitochondria are related to aging and many diseases, including cancer [73]. Mitochondria consist of four sub-compartments: the outer and inner membranes, the inter-membranes space, and the matrix. Mitochondrial morphology machinery is also modulated through post-translational modifications, such as phosphorylation, ubiquitination, and sumoylation [74]. Mitochondria contain their genome, which is composed of a small double-stranded circular DNA molecule (mtDNA). The human mitochondrial chromosome contains 37 genes (16,569 base pairs), including 13 that encode proteins of the respiratory chain; the remaining 24 genes encode for rRNA and tRNA, which are essential for the protein-synthesizing machinery of mitochondria. Nuclear genes encode for other mitochondrial proteins (enzymes and membrane transporters). On average, mitochondria contain five to seven genomes, which are stabilized by DNA-binding proteins that are different from the nuclear histones [75]. Given the limited activities of DNA repair and the high ROS production, mammalian mtDNA is more inclined to oxidative damage and is subjected to a higher mutation rate than nuclear DNA [76].

The critical targets of cadmium are the thiol groups (–SH) of cysteines present in proteins. The inactivation of the sulfhydryl groups of enzymes can produce several functional deficits in nuclei, the endoplasmic reticulum, and mitochondria. The toxic effect related to cadmium is mainly due to the blocking of the mitochondrial electron-transfer chain by impairing electron flow through complex III (cytochrome bc1 complex or ubiquinone: cytochrome c oxidoreductase). Cadmium inhibits ADP- and uncoupler-stimulated respiration and induces an ion permeability increase in the inner mitochondrial membrane by producing the opening of a mitochondrial permeability transition pore [77].

**Figure 2:**
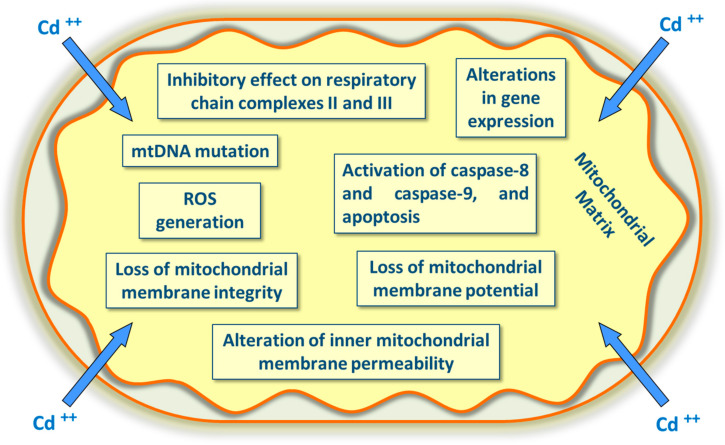
The main target in cadmium intoxication. Cadmium acts on mitochondria by inducing oxidative stress and generating reactive oxygen species (ROS), activating apoptosis, mutating mtDNA, altering gene expression, inhibiting respiratory chain complexes, reducing ATP synthesis, and altering the inner mitochondrial permeability.

Cadmium may alter many mitochondrial proteins’ activity (enzymes and transporter systems across the outer and inner membranes) by inhibiting respiratory chain enzymes [78], collapsing mitochondrial membrane potential, and swelling mitochondria, causing the inhibition of respiration [79]. Cadmium could directly increase permeability and decrease mitochondrial membrane potential, a condition that causes cytochrome C release with activation of the caspase pathway (Figure 2). Additionally, cadmium inhibits ATPase, lactate dehydrogenase (LDH), superoxide dismutase (SOD), and glutathione peroxidase (GPx) activities, enhancing the levels of ROS and lipid peroxidation [80]. Cadmium seems to be involved in Fenton reactions. Even though it does not act as a catalyst in the Fenton reaction, it can produce ROS by indirectly displacing an endogenous Fenton metal (e.g., Fe^2+^) from proteins, thus increasing the amount of free redox-active metals. Cadmium may alter the cellular redox status by reacting with exogenous and endogenous antioxidants, such as glutathione GSH [81]. Cadmium may damage mitochondria and not only interferes with Ca^2+^ signaling [82] but also increases mitochondrial ROS formation [83]. Cadmium ions act as xenobiotics by inhibiting the activity of complex II (succinate dehydrogenase) and complex III (cytochrome bc1 complex) of the electron transfer chain more than acting on complex I (NADH dehydrogenase or NADH: ubiquinone oxidoreductase) and complex IV (cytochrome oxidase). The principal site of ROS production seems to reside in the complex III, and ROS accumulation affects the potential of the mitochondrial membrane and activates a sequence of events, including apoptosis (a cellular self-destruction mechanism and a programmed and genetically encoded cell death) (Figure 2) [84].

There are two different apoptotic pathways: the extrinsic or death receptor-mediated pathway and the intrinsic or mitochondrial-mediated pathway; the two pathways are linked to each other and the molecules in one of the two pathways can influence those of the other. The extrinsic pathway triggers apoptosis in response to external stimuli, while the intrinsic pathway triggers apoptosis in response to internal stimuli, such as DNA damage. The mitochondrial pathway is related to toxic stimuli like ROS, UV radiation, ionizing radiation, Ca^2+^, and Cd^2+^, either indirectly through increases of Ca^2+^ and ROS. These stress stimuli lead to mitochondrial outer membrane permeabilization with the release of cytochrome C from the mitochondrial intermembrane space into the cytosol, thus activating caspase-8 and causing apoptosis. The intrinsic pathway is triggered by a cellular stress signal as DNA damage activates caspase-9. In addition to Cd toxicity, apoptosis can be induced by caspase-independent events or by Ca^2+^-calpain coupled processes. Excessive ROS production leads to a free radical attack of mitochondrial membrane phospholipids and mitochondrial membrane depolarization, which is a key step in the intrinsic pathway of apoptosis [85,86]. Each pathway activates its initiators, such as caspase-8 (extrinsic pathway) and caspase-9 (intrinsic pathway), which in turn activate the executioner caspase-3 and caspase-7. Caspases (cysteine-aspartic proteases) have proteolytic activity with cysteine in the active site that can cleave proteins after an aspartate residue. The execution pathway results in characteristic cytomorphological features, such as cell shrinkage, chromatin condensation, and the formation of cytoplasmic blebs and apoptotic bodies. Additionally, apoptotic cells exhibit protein cleavage, protein cross-linking DNA breakdown, and phagocytic recognition. In addition to apoptosis, excessive ROS production can lead to macromolecule oxidation with a free radical attack to phospholipids, disturbing the integrity of mitochondrial membranes, mitochondrial membrane depolarization, and mtDNA mutation (Figure 2) [85]. The major elements of mitochondrial apoptosis are represented by the generation of ROS, the alteration of mitochondrial membrane potential, and the activation of caspase-9. Cadmium induces apoptosis in vivo in several organs, including the liver and kidneys. In cultured cells, it elicits a cellular stress response that culminates in the activation of the mitochondrial apoptosis pathway [87]. In intact cells, Cd^2+^-induced mitochondrial damage with mitochondrial apoptosis is a late event, which occurs at least 15–24 h after Cd^2+^ exposure [82,88].

## 5. Cadmium and Metallothioneins

Metallothioneins (MTs) are ubiquitous low molecular weight proteins (MW 7–8 KDa). Their name is related to the occurrence of several divalent metal ions (Fe, Co, Ni, Cu, Zn, Ag, Cd, Hg, Pb) in the isolated material (metallo-) and the high –SH (cysteine) content of the protein (-thionein). Metallothionein was first identified in the kidney cortex of equines as a Cd-binding protein that is responsible for the accumulation of Cd in the tissues [89]. The mammalian MT is made up of 61–68 amino acid residues, 18–23 of which are cysteines. MT is a unique protein that is very rich in thiol groups. In mammals, four different MTs are expressed, which are termed MT1, MT2, MT3, and MT4. MT1 and MT2 are expressed in almost all tissues, while MT3 and MT4 are tissue-specific. The –SH groups of these metal proteins (apoproteins) can complex seven divalent cations, such as Zn^++^ and Cd^++^, or 12 monovalent metal ions [90]. A characteristic of mammalian MTs is the binding of divalent transition ions in two-metal –SH clusters, a M_3_S_9_ cluster in the N-terminal β-domain, and a M_4_S_11_ cluster in the C-terminal α-domain. In these mammalian MTs, the cations determine the tertiary structure of the protein, where these ions are immersed in the interior of the two domains. The MT chain wraps around these two clusters, binding up to several divalent cations with a tetrahedral geometry and with the –SH groups of twenty cysteines [91,92]. In mammals, MTs have been localized first in the cytoplasm of the cells, and then in lysosomes, mitochondria, and nuclei. The more important and physiological functions of MTs are the homeostasis of the essential metal ions Zn and Cu, the protection against Cd and other toxic metal cytotoxicities, and providing scavenging free radicals produced in oxidative stress [91]. The binding affinity of MTs for metal ions is metal-dependent, as seen in in vitro studies with common rat liver MTs. Last century, it was found that the relative order of MTs’ affinity for metals is Cd > Pb > Cu > Hg > Zn > Ag > Ni > Co.

Under normal physiological conditions, MTs bind zinc and copper, while the binding to cadmium depends on the accumulation of this metal ion in the kidney, which varies with age. In mammalian cells, Zn transporters, such as ZIP8 and ZIP14, have been reported to function as transporters for Mn(II) and Cd(II), contributing to the maintenance of Mn homeostasis and metallothionein-independent transports of Cd, respectively [72].

Regardless of the route of exposure, cadmium is efficiently retained in the tissues and remains accumulated throughout life. The body burden caused by Cd increases continuously during life and Cd accumulates the most in the liver and even more in the kidneys, which can contain up to 50% of the total body burden of this metal. After absorption, Cd in the blood is transported to the liver while bound to albumin, transferrin, and red blood cell membranes [93]. The accumulation of Cd in the liver and kidneys is due to the capability of these two organs to synthesize MTs, which are the precursor to Cd detoxification. Given the great ability of MT to bind heavy metals (including Cd), it has been suggested that this protein could play roles in both the intracellular fixation of trace elements Zn, Cu, and Cd when controlling the levels and neutralizing the harmful presence of toxic elements [94]. MTs may bind both physiological (Cu, Zn, Fe) and xenobiotic (Cd, Hg, Ni, Pb) heavy metals through four thiol groups of cysteines. Thus, cadmium is tetrahedrally coordinated to the sulfur, and therefore this bond is particularly strong. The stimulation of metallothionein by zinc probably explains the protective effect of this essential element toward Cd toxicity. MTs accumulate in hepatocytes as Cd-MT complexes, thus protecting the cell from toxic Cd ions. Most of these Cd-MT complexes are stored in the liver, even if small amounts of these complexes are released in the blood passing in the tubular fluid. Inside lysosomes of renal tubular cells, the Cd-MT complex is degraded to amino acids and free Cd ions. These released ions induce the synthesis of other MT proteins in the kidneys, where these complexes accumulate. The half-life of these Cd-complexes is low, and they dissociate in Cd-ions and free MTs [91]. In contrast, the half-life of Cd^++^ is very long (25–30 years) in the human body, where cadmium can unleash its toxic action [95,96]. MTs play an important role in the protection against metal toxicity and oxidative stress. Cysteine residues from MTs can also capture hydrogen peroxide and hydroxyl radicals. In this reaction, cysteines are oxidized to cystines and Cd ions, which are liberated from the cysteines. The biosynthesis of MTs is increased by several-fold throughout oxidative stress to protect the cells against cytotoxicity and DNA damage. In the human body, high levels of this protein are synthesized in the liver and kidney, and their production depends on the presence of dietary minerals, such as Zn, Cu, and Se, as well as the amino acids histidine and cysteine. After absorption, Cd is delivered to the liver via endogenous intestinal MTs. Then hepatic Cd-MT gradually redistributes this metal to the kidneys, which are the main target organ for Cd toxicity. After being released from the liver, Cd bound to MTs is distributed to other organs. In the kidney, Cd undergoes glomerular filtration, from where it is reabsorbed intracellularly from renal tubular cells. In the latter, Cd is cleaved from MTs and Cd^2+^ ions are re-excreted into the tubular fluid; finally, Cd is eliminated in the urine. An acidic medium, such as that present in lysosomes (pH 4.5–5.5), promotes the dissociation of Cd from a Cd-MT complex. Cd-MT shows very low toxicity to fetuses and the central nervous system compared with other heavy metals because it does not easily cross the placental and hemato-encephalic barriers. Cd is mainly eliminated through the urine, even if the amount of Cd excreted daily in urine is very low. This low excretion corresponds to the Cd biological half-life of more than 25–30 years [93,97].

## 6. Role of Oxidative Stress in Cadmium Toxicity

Cadmium is characterized by a strong cumulative effect in humans and its content in living organisms increases with diet and age [98]. Among the factors that determine the absorption and accumulation of this xenobiotic, the duration of uptake, the chemical form of the metal, and the diet should be included, as well as age, sex, and health conditions of the exposed people [99,100,101,102,103,104].

This heavy metal interferes with essential biometals like zinc, magnesium, selenium, calcium, and iron, altering their homeostasis and also disturbing their biological functions [53,55,105]. Gastrointestinal absorption of this metal is determined in the diet by the content of these essential elements, along with vitamins, polyphenols, antioxidants, and other active biomolecules. The enhanced intake of some bioelements may prevent the absorption and the toxic effects of Cd; at the same time, the deficiency of some of these biologically active substances can increase gastrointestinal absorption and the accumulation of Cd in the body [55,62,68,100,106].

**Figure 3:**
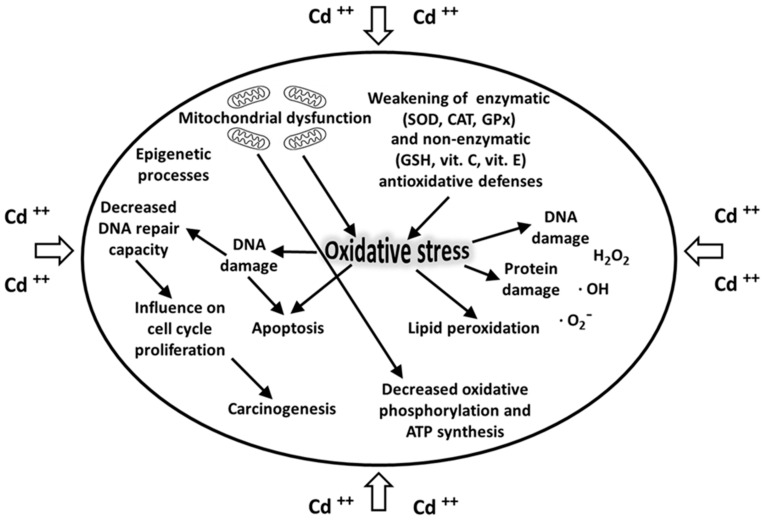
Cadmium acts on mitochondria, awakening the enzymatic and non-enzymatic antioxidative defenses. Cadmium induces oxidative stress, to resulting in the damage of proteins, lipids, and DNA. Cadmium decreases the activity of DNA repair enzymes, influencing cell cycle proliferation and stimulating carcinogenesis.

Cadmium is one of the most toxic heavy metals and its toxicity may be considered multidirectional. Cadmium ions show a high affinity for biological structures containing –SH groups (cysteine and glutathione GSH), as well as disulfide –S–S– groups (cystine and reduced glutathione GS-SG), causing disturbance of their functions. The bivalent cadmium cation is unable to generate free radicals directly; however, after cadmium exposure, there is increased production of ROS, namely superoxide radicals, hydrogen peroxide, and hydroxyl radicals. Cadmium induces oxidative stress [107] and the production of ROS that are normally balanced by the enzymatic (SOD, CAT, GPx) and non-enzymatic (GSH, vitamin C, vitamin E) antioxidative barriers [58,108]. The xenobiotic-induced oxidative stress results in the oxidation and damage of biologically important macromolecules, such as proteins, DNA, lipids, and cellular membrane phospholipids (Figure 3). Furthermore, by lowering the potential of mitochondrial membranes, cadmium disrupts the oxidative phosphorylation and synthesis of ATP [109].

The relevance in lipid peroxidation as a key determinant in cellular damage was confirmed by the effect of vitamin E alone or in combination with β-carotene, which reduced the harmful effects of cadmium [110].

It has also been demonstrated that this toxic metal may damage DNA and inhibit some DNA repair enzymes [111]. Cadmium also influences gene expression and stimulates carcinogenesis by disrupting cell signaling pathways (Figure 3). Finally, this toxic metal adversely affects the proliferation and differentiation of cells, thus contributing to their apoptosis and necrosis [53,112,113].

## 7. The Epigenetic Effects of Cadmium Exposure

Epigenetics refers to heritable changes in gene expression that do not involve a change in the nucleotide DNA sequence. The principal mechanisms that mediate epigenetic regulation of gene expression are DNA methylation; the post-translational modification of histones; small non-coding RNA molecules (microRNA, miRNA), which may interfere with gene transcription and/or translation; and the packaging of DNA around nucleosomes. The enzymes involved in epigenetic processes include DNA methyltransferase, histone methyltransferase, histone acetyltransferase, and histone deacetylase. These processes may be influenced by various environmental factors and their dysregulation is implicated in many diseases [114,115]. Recently, growing evidence has shown that heavy metals might exert their toxicity through miRNA. Aberrant alterations of the endogenous miRNA have been directly implicated in various pathophysiological conditions and signaling pathways, consequently leading to different types of cancer and disorders [116].

DNA methylation involves the covalent bond of a methyl group to the cytosine to form 5-methylcytosine in the presence of DNA methyltransferase and SAM (S-adenosyl methionine) as a methyl group donor. DNA methylation is involved in regulating cellular processes, including genomic imprinting, chromosome stability, and gene transcription. The N- and C-terminal tails of H_3_ and H_4_ histones undergo post-translational covalent reactions, such as methylation, acetylation, phosphorylation, ADP-ribosylation, ubiquitination, and sumoylation, which influence the chromatin structure and gene expression.

MiRNAs are small non-coding molecules of 20–25 nucleotides, which are involved in the post-transcriptional regulation of protein expression by degrading their target mRNA and/or inhibiting their translation, which is based on the degree of complementary base pairing. MiRNAs are transcribed from DNA but not translated in proteins. MiRNA’s main function is to down-regulate gene expression by interfering with messenger RNA (mRNA) functions [117,118].

Epigenetic changes can be triggered by environmental factors, such as the type of nutrition and exposure to xenobiotics, due to the dynamic state of the epigenome [119]. Cadmium exposure may alter gene expression profiles and change epigenetic components in three features: DNA methylation, histone post-translational modifications, and miRNAs. DNA methylation levels seem to be associated with the time of exposure to cadmium. In fact, Cd exposure for a short time (24 h–1 week) induces hypomethylation, while longer times (8–10 weeks) induce hypermethylation [120]. In vitro exposure to cadmium of TRL1215 rat liver cells for only one week inhibited DNA methyltransferase activity (up to 40%), ending in DNA hypomethylation, while longer exposures to Cd (10 weeks) of the same cells resulted in DNA hypermethylation because of an increase in the activity of DNA methyltransferase. Benbrahim-Tallaa et al. indicated that Cd-induced DNA hypermethylation occurred in association with the malignant transformation of human prostate epithelial cells [121].

MiRNAs are transcribed as ≈70-nucleotide precursors and subsequently processed by the Dicer endoribonuclease enzyme to produce miRNA (20–25 nucleotide products). In the nematode *Caenorhabditis elegans*, lin-4 was the first miRNA to be discovered [122]. Until now, several miRNAs have been discovered as epigenetic regulators in all organisms, such as vertebrates, plants, flies, worms, and viruses [123]. It should not be surprising that miRNAs regulate many processes, including metabolism, proliferation, differentiation, development, and cell death. Additionally, miRNA expression has been associated with oncogenesis, with some miRNA as oncogenes and others as tumor suppressors.

Ten years ago, Huang et al. constructed an miRNA library from rice seedlings exposed to Cd. The study of the library sequence showed 19 new miRNAs, where these cloned rice miRNAs had sequence conservation different from *Arabidopsis* and any other species. It is important to emphasize that most of these new rice miRNAs were up- or down-regulated in response to Cd exposure [118].

A more recent study demonstrated that Cd ions induced new miRNAs in NIH/3T3 cells [124]. Epigenetic aspects of the toxicity of cadmium telluride (CdTe) quantum dots attract more attention due to their ability to reprogram gene expression after the initial signal has been removed. Additionally, the involvement of epigenetic mechanisms in miRNA biogenesis suggests that miRNAs may act as participants in the cytotoxicity of CdTe quantum dots. According to SOLiD (sequencing by oligonucleotide ligation and detection) sequence results, the expression patterns of miRNAs are widely affected after CdTe quantum dot exposure, resulting in apoptosis-like cell death. Furthermore, the authors used the SOLiD sequence method to acquire the miRNA expression profiling in NIH/3T3 cells after exposure to CdTe quantum dots, Fe_2_O_3_ nanoparticles, and multi-walled carbon nanoparticles (MW-CNTs).

In a study on rice (*Oryza sativa*) conducted in the laboratory by using a microarray assay, Ding et al. identified 19 Cd-responsive miRNAs, six of which were further experimentally validated [125]. Target genes were also predicted for these Cd-responsive miRNAs, which encoded transcription factors, and proteins associated with metabolic processes and stress responses.

Since Cd exposure alters the gene expression profile along with changing epigenetic components, it is conceivable that epigenetic changes (including the silencing of DNA repair and tumor-suppressor genes) may be associated with carcinogenic effects.

## 8. Cadmium Carcinogenesis

Cadmium was classified by the International Agency for Research on Cancer (IARC) as a carcinogenic agent when inhaled, while there is not enough evidence in the case of oral ingestion to classify this xenobiotic as carcinogenic [15,25,126]. The major Cd-induced carcinogenic mechanisms include the induction of inflammatory processes, oxidative stress, ROS, epigenetics, attenuation of apoptosis, damage to DNA, decreased DNA repair capacity, alteration in gene expression, cell proliferation, and aberrant DNA methylation [111,112,118,127]. The presence of zinc finger motifs in steroid hormone receptors and other DNA-binding molecules offers targets for Cd binding and alteration in their biological function. The chemical similarity of Cd to Zn may be responsible for the toxic effects of Cd; in fact, the presence of Cd instead of Zn in the active center of histone demethylase has been described as a mechanism for Cd inhibition of this family of enzymes [128].

Oxidative stress is essential in cadmium toxicity [129]. It has been shown to promote tumor development through mutagenesis and effects on the cell cycle. A functional DNA repair system removes errors caused by metabolism and environmental carcinogens; however, inadequate repair mechanisms allow for the accumulation of damaged DNA, which promotes cancer [130]. Using this method, cadmium can inhibit DNA repair, including nucleotide and base excisions and mismatch [131]. The loss of a DNA repair mechanism allows for the accumulation of cells with damaged DNA, which following cell division, can produce carcinogenic mutations.

Some authors suggest that short-term Cd exposure inhibits DNA methyltransferase 1, inducing DNA hypomethylation, while chronic Cd exposure can activate DNA methyltransferase 1, resulting in hypermethylation [120,132,133]. In particular, Cd exerts an opposite effect depending on acute versus chronic exposure, as chronic heavy metal intoxication activates an adaptive mechanism to external stress. In fact, if the cells have been exposed for a short time to high doses of Cd (acute exposure), they may enter into apoptosis and have decreased metastatic potential, while chronic exposure leads to malignant transformation of normal epithelial cells as an adaptation process characterized by apoptosis resistance, increased invasion capacity, dedifferentiation, and self-renewal properties [134].

In Nagata et al., a combined odds ratio (OR) and corresponding 95% confidence interval (CI) were applied to assess the association between cadmium and breast cancer, where a positive association between urinary cadmium levels and the risk of breast cancer among Japanese women at a hospital in Gifu City was observed [135]. A total of 178 cases and 431 controls agreed to participate in the study and informed consent was obtained from each woman. Spot urine samples were collected from cases after surgery but before any cancer therapy; while from the controls, spot urine samples were collected at the data screening visit. Urinary Cd concentrations were expressed as urine cadmium/urine creatinine ratios. Women in the highest tertile of the creatinine-adjusted cadmium level (>2.620 μg/g) had a significantly higher OR of breast cancer than those in the lowest tertile (<1.674 μg/g) after controlling for variates (adjusted OR 6.05; 95% CI: 2.90, 12.62).

Nawrot et al. reported the results of an epidemiological study, conducted from 2006 to 2014, involving 20,459 study participants in Belgium and the USA [136]. The meta-analysis showed consistent evidence of total cancer and lung cancer in association with life-time environmental exposure to this toxic metal, as demonstrated by the urinary Cd concentration of creatinine (ranging from 0.25 to 0.93 μg/g). In their study, the authors suggested that Cd may be an important risk factor for lung cancer, where the pooled RR (relative risk) amounted to 1.68 (95% CI: 1.47–1.92).

The clinical studies of Peng et al. explored the relationship between the blood Cd burden and breast cancer in Chaoshan women without occupational exposure in Guangdong (China) [137]. Blood Cd concentrations were determined in the whole blood of 186 breast cancer cases and 139 controls. Blood Cd levels and proportions of blood Cd levels over 3 μg/L between cases and controls were compared. The breast cancer patients had a higher median concentration of blood Cd (2.28, interquartile range 1.57–3.15 μg/L) than the controls (1.77, interquartile range 1.34–2.57 μg/L). The proportion of breast cancer levels over 3 μg/L was 2.35 times higher in the breast cancer cases than that of the controls.

Peng et al. also investigated the relationship between the presence of Cd in blood and nasopharyngeal carcinomas (NPCs) in the Chaoshan population in southeast China [138]. A case–control study was conducted on 134 cases and 132 cancer-free controls from a cancer hospital in Chaoshan area (China). Clinical data information regarding lifetime styles, smoking, and drinking was collected from medical records. Blood Cd levels and over-limit ratios between cases and controls were compared. The Peng study results show that the median concentration of blood Cd in cases (3.84, interquartile range 2.21–6.10) was significantly higher than that of controls (2.28, interquartile range 1.79–3.45). The over-limit ratio (>5 μg/L) in cases (35.1%) was also higher than that in controls (13.6%). Smokers tended to have high levels of Cd burden. According to this study, Cd seems to be a risk factor of NPC, and Cd exposure may promote the occurrence and development of NPC.

Additionally, recent epidemiological data indicate that exposure to this toxic metal in the environment may be related to cancers of the prostate [139], urinary bladder [140], pancreas [141], and kidneys [142].

## 9. Phytoremediation, Nanoparticles and Microbial Fermentation against Cadmium Toxicity

Nowadays, modernization, industrialization, and fertilization are the major cause of contamination in the ecosystem, which is a serious issue all over the world. Toxic heavy metal pollution of water and soils is a major environmental problem and most conventional approaches do not provide acceptable solutions; in any case, these solutions are invasive and expensive. Sewage sludge is often used as a soil additive to improve the soil’s physical, chemical, and biochemical properties, and as a good source of plant nutrients. This sludge contains increasing amounts of heavy toxic metals, pesticides, detergents, and various organic materials [143].

Cadmium has no biological function in plant growth but is readily accumulated in all plants and edible parts of crops through the metabolic pathway of essential nutrients for the plants, such as Zn and Fe. Thus, by entering human beings’ and animals’ food chains, it may cause a wide variety of acute and toxic effects [144].

Additionally, cadmium inhibits root and shoot growth, causing a decreased uptake of nutrients. In plants, the toxicity of Cd may cause a decrease in the chlorophyll content, rate of photosynthesis, stomatal behavior, transpiration rate and relative water content, leaf necrosis, inhibition of the activity of enzymes, and an alteration in enzymes of the antioxidant defense system and nitrogen metabolism, which ultimately reduces plant growth and development. The use of plants to remediate polluted soils has been adopted with great success. Phytoremediation and nanoparticles are used as ecofriendly techniques and can be applied to both inorganic and organic pollutants, which are present in the water, soil, and air [145].

The toxic nature of heavy metals, which accumulate in the environment, has caused important health problems to human beings. Additionally, the removal of heavy metals is a necessary step to provide clean water resources for the population. Reverse osmosis and membrane separation, ion exchange, and chemical precipitation are some of the common techniques used for the removal from aqueous solutions of heavy metals. These methods are the most expensive, requiring high amounts of energy or large quantities of chemicals.

The phytoremediation of heavy metals (Hg, Pb, Ni, Co, Fe, Cd, etc.) through the use of plants is increasing in the scientific literature [146]. Jadia and Fulekar studied the phytoremediation of heavy metals (including cadmium) by sunflower (*Helianthus annuus* L.) in soil-vermicompost media [147]. The heavy metals (Cd, Cu, Ni, Pb) were dosed at various concentrations (0, 10, 20, 40, and 50 ppm) separately in soil/vermiculite media (3:1) in a pot experiment. At lower concentrations of heavy metals (0, 10, 20 ppm), a stimulation of the root and shoot length and an increase in the biomass of the sunflower were observed. In contrast, at higher concentrations of xenobiotics (40 and 50 ppm), the seed germination and root and shoot growth were significantly affected. The research study indicates that sunflower is capable to remediate heavy metals at all concentrations ranging from 0 to 50 ppm. Vermicompost can be used to remediate metal-contaminated soil, as well as serving as a natural fertilizer in soil and helping the growth of sunflower plants. Then, Al-Dhaibani et al. studied the ability of sunflower (*Helianthus annuus* L.) to remove Cd from a soil polluted with sewage sludge at the field station of King Abdulaziz University (Jeddah, Saudi Arabia) [148]. The mobility of Cd from root to shoot, as well as the seeds, was evaluated. As the xenobiotic metal concentration in the sewage sludge increased, the cadmium in each plant part significantly increased. Sunflower plants may remove around 57%–72% cadmium from the initial polluted soil concentration of metal.

Recently, a statistical experimental strategy was used to optimize the Cd adsorption using chemically treated stems of *Helianthus annuus* L. [149]. Goswami and Das studied cadmium phytoremediation by Indian mustard *Brassica uncea* [150]. These researchers used CdCl_2_ at concentrations of 0, 25, 50, 100, 200, and 400 mg/kg for 21 days, and estimated the cadmium concentrations in the roots, shoots, and leaves using atomic absorption spectroscopy. They found that the plant showed a high Cd tolerance of up to 400 mg/kg with a general decline in the root and shoot lengths, as well as leaf chlorophyll and carotenoid contents. The highest shoot and root (10,791 μg/g dry wt and 9602 μg/g dry wt, respectively) cadmium accumulation was achieved with a 200 mg/kg cadmium treatment, while the maximum leaf cadmium accumulation was 10,071.6 μg/g dry wt at 100 mg/kg cadmium, after 21 days of treatment. These results show the ability of *Brassica uncea* to remove Cd from soil.

Iori and collaborators investigated the physiological and biochemical parameters related to Cd tolerance and accumulation in eucalypt plants. Two clones (Velino ex 7 and Viglio ex 358) of *Eucalyptus camaldulensis* Dehnh. × *E. globulus* subsp. *bicostata* (Maiden, Blakely, and Simm) B Kirkp were exposed to 50 μM CdSO_4_ under hydroponics for three weeks [151]. Cadmium accumulation occurred in the roots of both clones with a poor amount of the metal in the aerial parts. The phytoremediation efficiency highlighted a good Cd removal ability, especially for the Velino clone.

Bora and Dutta [152] and Stietiya and Wang [153] used nanoparticles of TiO_2_ and Al_2_O_3,_ respectively, to very efficiently remove Cd from wastewater and a soil solution due to the high specific surface area. Stietiya and Wang added phosphate, citrate, and humic acid to affect the efficiency of Al_2_O_3_ nanoparticles regarding the adsorption of Cd [153]. As a result, phosphate was shown to be the most effective among the three ligands in enhancing Cd adsorption.

The study of Al-Qahtani aimed at synthesizing zero-valent silver nanoparticles to remove Cd from aqueous solution [154]. Ag-nanoparticles were prepared using AgNO_3_ and an extract of *Ficus benjamina* leaves. These silver nanoparticles have the advantage of high reactivity, easy preparation, and low cost. The removal of Cd increased when the pH increased from 1 to 6, contact time increased from 5 to 40 min, and the quantity of biosorbent increased.

Synthesized alumina nanoparticles and alumina nanoparticles modified with negatively charged glycerol were tested for high-efficiency Cd removal from simulated groundwater [155]. The maximum removal efficiencies of both synthesized and glycerol-modified alumina nanoparticles were about 99%–100%. Cadmium removal using synthesized and glycerol-modified alumina nanoparticles were due to ion exchange and electrostatic attraction. However, the stronger bond between cadmium and glycerol-modified alumina nanoparticles makes the latter preferable in the process of removing the metal from contaminated water.

The presence of cadmium in food is variable and depends on the geographical location, the bioavailability of cadmium from the soil, crop genetics, agronomic practices used, and postharvest operations [156].

Recently, microbial fermentation has been demonstrated to be a promising method for removing Cd from food [157]. The removal of Cd from rice using *Lactobacillus plantarum* fermentation has been reported [158]. Then, Zhu et al. studied the optimal conditions for reducing Cd in rice noodles using mixed *Lactobacillus* fermentation. The authors demonstrated that this fermentation can efficiently reduce an excess concentration of Cd in rice flour, improve its physicochemical properties, and promote the quality property improvement of rice noodles made by it [159].

## 10. Conclusions

Cadmium is one of the most toxic elements that human beings can be exposed to at work or in the environment and has no known physiological role in mammals. Human exposure to Cd can occur through food, water, and the inhalation of cigarettes. Once absorbed, Cd is efficiently retained in the human body, where it accumulates throughout life with a half-life of 25–30 years. Recent studies indicate that cadmium is capable of inducing epigenetic changes in mammalian cells. Since aberrant epigenetics play a decisive role in the development of various cancers and chronic diseases, Cd may cause pathogenic risks via epigenetic mechanisms. Several epidemiological and experimental data indicate that chronic exposure to cadmium in human beings can be associated with carcinogenesis, primarily in the lung, but also in the prostate, kidneys, breast, urinary bladder, nasopharynx, pancreas, and hematopoietic system. Once absorbed in the liver and kidneys, cadmium induces the synthesis of metallothioneins, which are small metal-binding proteins that are very rich in cysteines. The major physiological functions of metallothioneins include the homeostasis of essential metal Zn and Cu, protection against the cytotoxicity of Cd and other toxic metals, and scavenging free oxygen radicals generated in oxidative stress. Cd^++^ ions are unable to generate free radicals directly; however, after cadmium exposure, there is an increased production of ROS, resulting in oxidative damage to various molecules, such as nucleic acid, enzymes, and membrane phospholipids. Additionally, Cd induces an increase in ROS formation, which in turn induces DNA damage and interaction with DNA repair mechanisms. Cd exerts its toxic effect mainly by blocking the mitochondrial electron-transfer chain by impairing electron flow through complex III (cytochrome bc1 complex). Excessive ROS production can lead to macromolecule oxidation, mitochondrial membrane depolarization, mtDNA mutation, and apoptosis. Furthermore, by lowering the potential of mitochondrial membranes, Cd disrupts the oxidative phosphorylation and decreases the synthesis of ATP. Cd induces oxidative stress and ROS, which are normally balanced by enzymatic (SOD, CAT, GPx) and non-enzymatic (GSH, vitamin C, vitamin E) antioxidant barriers.

## 11. Future Perspectives

The pollution of soil, water, and air is the most outstanding outcome of the evolution of our society. Soil pollution due to heavy metals, such as Hg, Pb, Ni, and Cd, is very harmful to live organisms. This type of pollution is mainly caused by anthropogenic activities, such as vehicle traffic, burning of fossil fuels, mining and metallurgical activities, and disposal sludge. The reclamation of heavy-metal-polluted soils can be achieved with different methods and technologies depending on the type of heavy metal, its speciation, the soil properties, and the economic cost. Biologically active compounds, such as polyphenols, melatonin, carotenoids, L-carnitine, and coenzyme Q10, can be used to alleviate the harmful health effects of cadmium exposure. An interesting alternative to reducing pollution by heavy metals is the adoption of biological methods, which consist of using plants or nanoparticles that accumulate heavy metals. This process of metal absorption and accumulation is called phytoremediation. This technology is a low-cost, effective, and ecofriendly method for removing pollutants such as heavy metals from contaminated soil, sludge, and groundwater. Recently, a microbial fermentation method has been used to reduce cadmium levels in contaminated rice. Studies about the removal of Cd need further investigation to reduce the effects of this heavy metal’s toxicity. 

## Figures and Tables

**Table 1:** Physical and chemical properties of cadmium.

Atomic number	48
Atomic weight	112.41 u
Atomic radius	155 pm
Electronic configuration	[Kr]4d^10^5s^2^
Melting point	321.07 °C
Boiling point	767.3 °C
Density at 20 °C	8.65 g/cm^3^
Reduction potential Cd^2+^ + 2e^−^ → Cd(s)	−0.40 E°
Heat of fusion	6.21 kJ/mol
Heat of vaporization	99.6 kJ/mol
Electronegativity (Pauling scale)	1.69
First ionization energy	867.8 kJ/mol
Second ionization energy	1631.4 kJ/mol

**Table 2:** Polyphenolic substances prevent harmful health effects caused by exposure to cadmium. Polyphenols constitute one of the most common molecules present in plants, with a wide range of biological activities related to their antioxidant actions. These compounds have at least one aromatic ring with one or more active hydroxyl groups. This table provides some relevant substances and their polyphenol contents (mg/100 g or mg/100 mL) are reported.

Polyphenolic Substances	mg Polyphenol/100 g
Cloves	15,200
Peppermint	11,960
Anise	5460
Cocoa powder	3450
Dark chocolate	1660
*Fruits*	
Black chokeberries	1700
Highbush blueberries	560
Blackberries	260
Strawberries	235
Red raspberries	215
Black currants	758
Plums	377
Sweet cherries	274
Apples	136
*Nuts*	
Hazelnuts	495
Walnuts	28
Almonds	187
Pecans	493
*Vegetables*	
Green olives	346
Artichokes	260
Red onions	168
Chicory	166
Spinach	120
Extra-virgin olive oil	62
*Beverages*	
Coffee	214
Black tea	102
Red wine	100
Green tea	90
